# Ecological and Embodied Assessment of Inhibitory Control Using a VR Stroop Task in Cognitively Healthy Older Adults: A Cross-Sectional Study

**DOI:** 10.3390/healthcare14070866

**Published:** 2026-03-27

**Authors:** Si-An Lee, Jin-Hyuck Park

**Affiliations:** 1 Department of ICT Convergence, The Graduate School, Soonchunhyang University, Asan 31538, Republic of Korea; iop5213@naver.com; 2 Department of Occupational Therapy, College of Medical Science, Soonchunhyang University, Asan 31538, Republic of Korea

**Keywords:** executive function, inhibitory control, virtual reality, stroop, embodied cognition, kinematics, ecological validity, older adults

## Abstract

**Background/Objectives**: This study examined the concurrent validity and test–retest reliability of the novel virtual reality-based Stroop test (VRST), developed based on the principles of embodied cognition. The VRST simulates a clothing-sorting task to assess inhibitory control using cognitive and behavioral (kinematic) measures. **Methods**: A total of 224 cognitively healthy older adults (mean age = 71.51 years) completed the VRST and a traditional Stroop test in randomized order. The VRST implemented a fixed-difficulty design consisting of 30 incongruent trials, where participants were required to sort virtual objects by their semantic category while ignoring conflicting color cues. The task duration ranged from approximately 1 to 3 min. The VRST assessed task completion time, error count, 3D movement distance, and hesitation latency. Test–retest reliability was examined after two weeks. Concurrent validity was analyzed via Pearson correlation coefficients with traditional Stroop metrics. Test–retest reliability was assessed using intraclass correlation coefficients (ICCs). **Results**: VRST performance metrics showed significant correlations with traditional Stroop completion time: task completion time (*r* = 0.821; *p* < 0.001), movement distance (*r* = 0.801; *p* < 0.001), and hesitation latency (*r* = 0.784; *p* < 0.001), indicating good concurrent validity. No significant correlations were observed for error counts. Test–retest analysis showed high reliability for completion time (ICC > 0.9; *p* < 0.001), movement distance (ICC > 0.9; *p* < 0.001), and hesitation latency (ICC > 0.9; *p* < 0.001), but not for error count. These findings suggest that the VRST provides reliable and ecologically grounded behavioral indicators of inhibitory control. **Conclusions**: This preliminary study supports the VRST as a valid and reliable measure of inhibitory control in healthy older adults. By combining kinematic data with realistic task contexts, the VRST extends executive function assessment beyond traditional methods. Although limited to non-clinical populations, the findings suggest its utility for detecting subtle variations in executive functioning during healthy aging, warranting further investigation across broader cognitive profiles.

## 1. Introduction

Inhibitory control is a fundamental aspect of cognitive function, enabling individuals to suppress inappropriate or irrelevant stimuli, thoughts, or behaviors. It is critical for maintaining goal-directed behavior and regulating impulsive responses [1]. This ability is frequently employed in daily life, particularly when individuals need to regulate their emotional impulses. However, healthy older adults often experience a decline in inhibitory control, which can lead to cognitive and behavioral dysfunctions and is associated with mental health conditions such as depression [2]. Clinical populations, including those with schizophrenia and autism spectrum disorders, also exhibit notable deficits in inhibitory control [3].

To assess inhibitory control, various neuropsychological tools have been developed, with the Stroop test being one of the most widely used. This task involves presenting incongruent stimuli—such as mismatched color and word cues—to induce cognitive interference, requiring the suppression of automatic responses. The performance is typically measured using response time and accuracy, offering insights into an individual’s level of inhibitory control [4]. In addition to performance-based tasks, some self-report questionnaires are also used to evaluate inhibitory capacity across diverse populations [5].

Despite the range of tools available, conventional tests primarily focus on observable behaviors or self-reports, limiting their ability to capture the dynamic interaction between biological, psychological, and sociocultural factors [6]. Furthermore, it is often difficult to determine whether a poor result is due to impaired interference control or broader deficits in behavioral regulation. In clinical settings, inconsistent findings from neuropsychological tests among individuals with cognitive impairment have further highlighted the need for alternative assessment methods that reflect the complexity of executive functioning [7].

Unfortunately, conventional paper-and-pencil tests widely used in clinical settings also suffer from limited ecological validity, as the structured tasks often do not resemble real-life situations, thereby restricting the generalizability of findings [8]. In response to these limitations, virtual reality (VR) technologies have emerged as a promising approach [9]. VR provides immersive environments that closely mimic real-world conditions, offering a more ecologically valid context in which to assess cognitive functioning [10]. Recent efforts to adapt the Stroop task into VR have successfully enhanced environmental realism; for instance, the virtual reality cognitive assessment utilized a virtual classroom to evaluate inhibitory control [11], and more recent paradigms have explored VR-based assessments to distinguish cognitive status through response-based metrics [12]. Beyond realism, the interaction between sensory and motor experiences in VR aligns with the theoretical framework of embodied cognition [13]. According to this perspective, cognition is not confined to internal mental processes but arises from interactions between the brain, body, and environment [14]. Inhibitory control, as a component of executive function, may thus be more accurately evaluated through real-time interactions within immersive tasks [14]. Previous studies have demonstrated that kinematic data captured during VR tasks can reflect specific cognitive domains such as executive function [11]. The methodological and conceptual novelty of our VR-based Stroop Test (VRST) lies in its departure from the static response systems seen in previous work [10,11]. Unlike existing VR-Stroop assessments that measure only the terminal response time, the VRST analyzes interference-in-motion by decomposing 3-dimensional (3D) movement trajectories into high-resolution kinematic markers.

Targeting cognitively healthy older adults is vital for identifying the preclinical window of executive decline [15]. While this population maintains general functional independence, subtle erosion of inhibitory control often manifests as a precursor to more significant cognitive impairment. However, traditional paper-and-pencil or computerized assessments often encounter ceiling effects in high-functioning seniors, they may fail to capture early, sub-clinical lapses in inhibitory control [16]. Extracting continuous kinematic metrics within this specific demographic provides a more sensitive measure of cognitive-motor integration by detecting nuanced behavioral fluctuations that remain invisible in binary correct/incorrect scores [17]. Establishing these normative benchmarks in healthy cohorts is a prerequisite for distinguishing successful aging from pathological decline and developing timely preventive interventions [18]. This suggests that carefully designed VR tasks may not only replicate real-world demands but also extract rich behavioral metrics that serve as high-resolution digital biomarkers for the early detection of inhibitory control.

Despite its potential, most current VR-based tools merely replicate traditional paper-based tasks in a virtual space without leveraging the full capacity of embodied cognition frameworks [19]. Specifically, while many existing VR-Stroop paradigms have integrated ecological context, they often treat motor output as a simple binary outcome, which fail to capture the continuous flow of cognitive interference into physical action. In contrast, the VRST captures embodied kinematic features by requiring participants to perform goal-directed reaching movements in a 3D workspace. This approach allows for the measurement of subtle motoric hesitations and trajectory deviations, providing a more granular assessment of how inhibitory conflict manifests in real-time motor behavior [20]. By leveraging these dynamic metrics, the VRST offers a more sensitive digital biomarker of inhibitory control than traditional VR adaptations that focus solely on discrete performance outcomes. In this regards, our study addresses a critical gap in the literature, as few studies have examined how VR-based tasks that simulate real-life actions and measure physical interaction dynamics can provide more valid assessments of inhibitory function [19].

Although VR-derived behavioral approaches have shown promise for advancing early detection of cognitive change, several limitations continue to restrict their clinical applicability. Many existing studies have assessed performance within virtual simulations of everyday tasks, such as kiosk use, extracting behavioral features to distinguish cognitive status [21,22]; however, these immersive environments often lack explicit cognitive targeting, particularly for domains such as inhibitory control, making it difficult to determine whether performance differences reflect specific cognitive impairments or general task demands. Furthermore, because these tasks simultaneously engage multiple processes, including memory, attention, and planning, the interpretation of outcomes becomes complex, reducing construct specificity [23,24].

Therefore, this study aimed to examine the concurrent validity and test–retest reliability of a novel virtual reality-based Stroop test (VRST) developed to assess inhibitory control. The task was specifically designed to engage inhibitory regulation within an ecologically valid virtual environment, ensuring that cognitive demands align with real-world contexts rather than abstract laboratory conditions. The test simulates a common daily task—organizing clothing items—to measure Stroop interference effects through both cognitive and behavioral metrics. By evaluating both response accuracy and embodied kinematic characteristics (e.g., movement distance and latency), this study seeks to provide a theoretical contribution by demonstrating that cognitive-motor integration can serve as a sensitive digital biomarker for inhibitory control. By evaluating both response accuracy and movement characteristics, this study seeks to determine whether VR-based tasks can offer a more comprehensive assessment of executive function in healthy older adults.

## 2. Materials and Methods

Although a portion of the dataset used in this study overlaps with data collected during an earlier investigation on the use of a VR–based Stroop task for detecting mild cognitive impairment [25], the aims and analytical scope of the two studies are fundamentally different. As summarized in Table 1, while the previous study utilized a combined cohort of both healthy older adults and individuals with mild cognitive impairment (MCI) for group comparisons to establish the diagnostic utility of the VRST’s kinematic markers, the current study exclusively utilizes the data from the 224 cognitively healthy participants to validate the psychometric properties of the tool itself. Furthermore, whereas the earlier work was a cross-sectional analysis of a single-session performance, the present study introduces a longitudinal stability of these embodied metrics through two-week test–retest reliability and confirms their concurrent validity against the traditional paper-based Stroop test. Thus, while the kinematic variables are shared, the current study provides the essential psychometric foundation—ensuring these markers are stable and valid—which was no addressed in the previous work. This makes the research questions, methodological emphasis, and contributions of the current study entirely distinct.

### 2.1. Participants

A total of 224 healthy older adults were recruited from senior welfare centers and adult day care facilities in Seoul and Asan, South Korea. The inclusion criteria were as follows: (1) intact global cognitive function, as determined by a score above the cutoff on the Korean version of the Mini-Mental State Examination (MMSE-K); (2) normal color vision; (3) no impairment in upper or lower limb muscle strength or joint range of motion; and (4) no symptoms of motion sickness while using a head-mounted display (HMD). Exclusion criteria included: (1) clinically diagnosed dementia; (2) neurological or psychiatric disorders such as stroke or depression; (3) uncorrected visual or auditory impairments; and (4) refusal or inability to wear an HMD due to discomfort or physical constraints.

### 2.2. Procedure

Participants completed two Stroop tests in a randomized order determined by a computer-generated random sequence: the VRST and the traditional paper-based Stroop test. This counterbalancing was employed to mitigate potential learning or fatigue effects that could arise from a fixed testing sequence. Prior to VRST administration, participants were guided through a standardized tutorial and practice session of at least 10 min to familiarize them with the VR interface, including wearing the HMD and using the hand controller. This session continued until participants demonstrated proficiency in 6-DoF manipulation to ensure that results reflected cognitive performance rather than technical unfamiliarity. Individual HMD strap settings and focal lengths were adjusted for comfort and clarity. After completing the tutorial, participants were given a 10 min break and were then seated with both feet flat on the floor. The VRST was conducted in a quiet, private room, with the examiner standing nearby to ensure safety without interfering with the task.

The VRST was implemented using the HTC VIVE system (HTC Corp., Taoyuan, Taiwan), which utilizes SteamVR Tracking 1.0 (Lighthouse base stations) to ensure high-precision kinematic data acquisition. The HMD provides a resolution of 1080 × 1200 pixels per eye, a 110° field of view, and a 90 Hz refresh rate. The task environment was custom-developed by research team using the Unity 3D engine platform (Unity Technologies, San Francisco, CA, USA). The system was operated on a PC running Windows 10, equipped with an Intel Core i7-10700 CPU and 16 GB of RAM.

For movement tracking, 6-DoF (six-degrees-of-freedom) handheld controllers were used, offering sub-millimeter tracking accuracy (approximately < 1 mm). All behavioral and kinematic data, including 3D coordinates and timestamps, were captured at a sampling rate of 90 Hz, providing high temporal and spatial resolution for the analysis of movement distance and hesitation latency. All participants were instructed to use their dominant hand to control the device.

**Figure 1:**
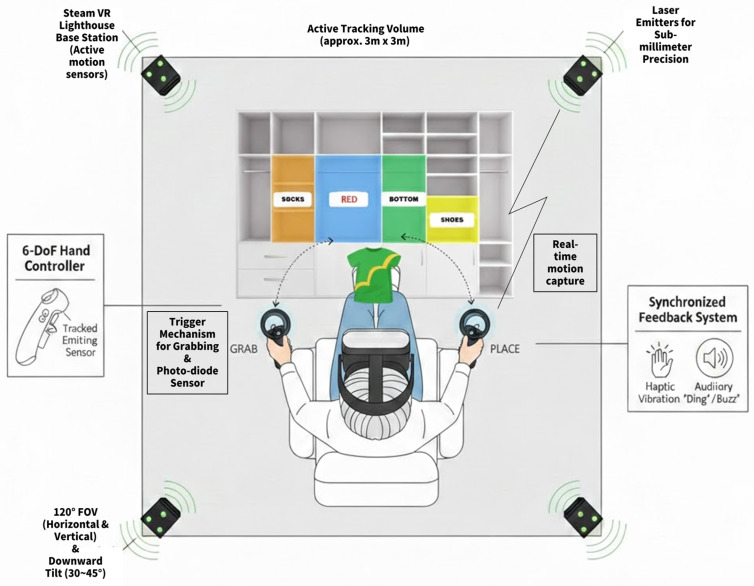
Schematic of the VRST experimental setup and participant interaction.

**Figure 2:**
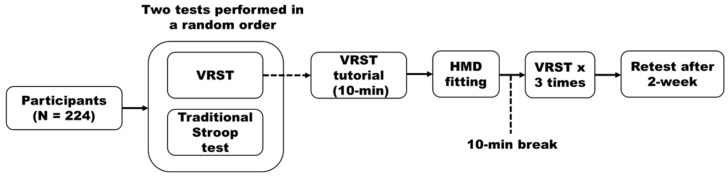
The flow diagram of the procedure of this study. VRSR: Virtual reality-based Stroop test.

The VRST is implemented as a functional task set in a virtual dressing room. Each session of the VRST lasted approximately 2 min and was repeated three times, with a 30 s break between sessions. The task comprises a total of 30 trials per session, consisting of 30 distinct incongruent stimuli (e.g., the word ‘Red’ on a blue shirt). To prevent predictability and control for order effects, the software’s algorithms fully randomized the sequence of these 30 stimuli for every participant and every session. In each trial, a 3D clothing item appears on a central hanger; participants must identify the font color of the word printed on the clothing, ignore the semantic meaning, and grab the item using the 6-DoF controller’s trigger. They then place the item into one of four color-coded storage boxes located at a 1.5 m distance within the virtual space. To ensure standardized delivery, the system provides immediate haptic vibration upon grabbing and auditory feedback (a soft ‘ding’ or ‘buzz’) to indicate correct or incorrect sorting (Figure 1). The VRST was repeated 2 weeks later under identical conditions for test–retest reliability analysis (Figure 2). The traditional Stroop test was administered at a desk using printed stimuli. Participants were seated, and the examiner sat adjacent to them, recording response time and errors using a stopwatch and answer sheet.

### 2.3. Outcome Measures

**Figure 3:**
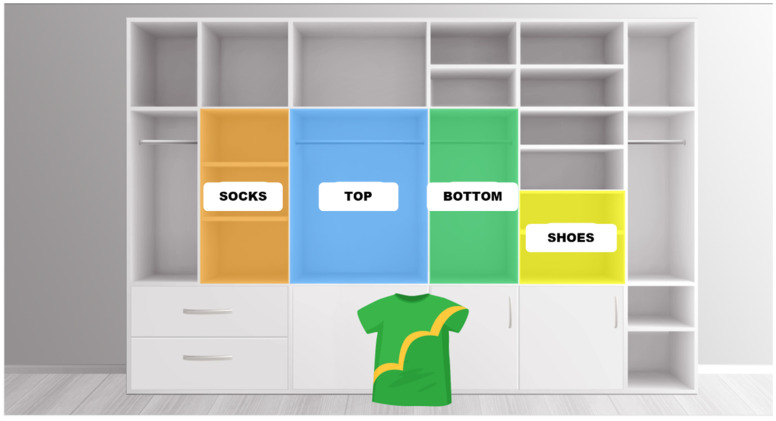
The screenshot of the VRST: participants used embodied interaction to sort incongruently labeled clothing items into matching category boxes.

The VRST was developed specifically for this study based on embodied cognition principles (Figure 3). The construction of the VRST followed a systematic digital framework development process, consistent with recent methodological approaches that emphasize multi-level indicator design and expert validation [26]. To ensure the rigor and validity of the tool, the task was developed through a three-stage process: (1) identification of core inhibitory control components, (2) mapping these components to a multi-level indicator system comprising both cognitive and embodied kinematic metrics, and (3) content validation by a panel of experts in occupational therapy and cognitive neuroscience.

This expert review ensured that the virtual clothing-sorting task accurately simulated the cognitive demands of the Reverse Stroop effect within an ecologically valid ADL (activities of daily living) context. Unlike the traditional color-naming Stroop, the VRST operationalized interference by requiring participants to prioritize semantic category over salient visual color. Stroop interference was induced by displaying each category word in a font color that conflicted with the target destination. Participants wore an HMD and used a handheld controller to move each stimulus into a matching virtual storage box labeled with the correct category (not color). The colors of both the stimuli and the storage boxes were randomized at each test to minimize learning effects.

The VRST developed for this study produced four behavioral outcome metrics, designed as a multi-level assessment system to capture both the outcome and the process of inhibitory control. These measures were not arbitrarily selected but represent the full set of behavioral data captured via the VR system, each aligning with established cognitive theories. First, the total completion time was recorded, representing the duration required to correctly sort all 30 stimuli. It is interpreted as an indicator of general processing speed and attentional control, reflecting cognitive efficiency as conceptualized in Sternberg’s memory scanning paradigm [27]. Second, the number of errors was calculated based on how many items were placed into incorrect storage boxes. Third, the three-dimensional movement distance of the controller was measured across the x, y, and z axes. This metric reflects inefficiencies in motor planning and spatial control often modeled by motor–cognitive integration frameworks such as the diffusion decision model [28]. Lastly, hesitation latency was captured, defined as the time interval between pressing the controller button and the initiation of object movement. This indicates delays in response selection and initiation, consistent with the controlled processing deficits described in Schneider and Shiffrin’s dual-process theory [29]. By integrating these cognitive (accuracy, time) and kinematic (distance, hesitation) indicators, the VRST framework provides a comprehensive evaluation of how cognitive interference manifests in physical action, a level of detail often missing in traditional single-metric assessments. These four variables were consistently measured across both the initial assessment and the follow-up session conducted two weeks later. Two weeks later, a subsample of 30 participants repeated the same VRST task under identical conditions to assess test–retest reliability. To minimize selection bias, these participants were selected from the pool of individuals who expressed willingness to participate in a follow-up session using a simple random selection process. Specifically, each consenting participants was assigned a unique identification number, and a computer-based random number generator was employed to select the final 30 participants.

The traditional Stroop test employed the word–color interference condition using the Korean version of the Stroop task. Participants were instructed to name the ink color of 112 color-word stimuli printed on an A4 sheet, in which the word meaning and the ink color were always incongruent. Testing continued until all items were completed or 2 min had elapsed. The number of correct responses and errors was recorded by an examiner using a stopwatch [30].

### 2.4. Statistical Analysis

All statistical analyses were performed using SPSS version 22.0. Descriptive statistics were computed for demographic variables. Pearson correlation coefficients were used to assess the concurrent validity between the VRST (four outcome variables) and the traditional Stroop test (completion time and error count). This bivariate correlation approach was selected as it provides a direct and standardized measure of concurrent validity, serving as a fundamental psychometric step in validating a new assessment tool against a recognized gold standard [31]. To strength the statistical rigor of the validity report, 95% confidence interval (CIs) were calculated for all Pearson correlation coefficients. The magnitude of the effect sizes for Pearson’s r was interpreted based on Cohen’s criteria: small (0.10–0.29), medium (0.30–0.49), and large (≥0.50) [32]. Test–retest reliability of the VRST was analyzed using intraclass correlation coefficients (ICCs) based on a subset of 30 participants who repeated the task with a 2-week interval between test sessions. A *p*-value of less than 0.05 was considered statistically significant.

## 3. Results

### 3.1. Participant Characteristics

The mean age of the participants was 71.51 years (SD 2.89), and the sample included 103 males and 121 females. The average years of education was 5.94 years. Fine motor function, as measured by the Grooved Pegboard Test, yielded an average completion time of 73.39 s for the preferred hand. Cognitive screening scores based on the MMSE-K indicated preserved cognitive functioning, with a mean score of 26.77 (Table 2).

### 3.2. Performance on VR-Based and Traditional Stroop Tests

For the VRST, the average task completion time was 72.65 s, and participants made an average of 3.32 errors. The mean total movement distance of the controller in three-dimensional space (x, y, z axes) was 22.43 cm. The hesitation latency—the time interval between pressing the controller button and initiating movement—averaged 5.17 s (Table 2). In the traditional paper-based Stroop test, the average task completion time was 72.54 s, and the mean number of errors was 5.47 (Table 3).

### 3.3. Concurrent Validity of the VR-Based Stroop Test

Correlation analyses were conducted to examine the concurrent validity of the VRST by comparing its outcome variables with those from the traditional Stroop test. A statistically significant positive correlation was observed between the VRST completion time and the traditional Stroop test completion time (*r* = 0.821, 95% CI = 0.773 to 0.860, *p* < 0.01), representing a large effect size. This indicates that participants who performed faster on the VRST also tended to complete the traditional test more quickly.

The total movement distance of the controller during the VRST was also significantly correlated with the traditional Stroop completion time (*r* = 0.801, 95% CI = 0.749 to 0.843, *p* < 0.001, large effect size). Similarly, hesitation latency during the VRST was significantly correlated with the completion time of the traditional Stroop test (*r* = 0.84, 95% CI = 0.728 to 0.829, *p* < 0.001, large effect size). These results suggest that greater movement efficiency and shorter hesitation latency in the VR environment were associated with faster cognitive performance in the traditional setting.

No statistically significant correlations were found between any VRST variables and the number of errors in the traditional Stroop test. This indicates that, except for error count, the VR-based Stroop test demonstrates good concurrent validity (Table 4).

### 3.4. Test–Retest Reliability of the VR-Based Stroop Test

**Figure 4:**
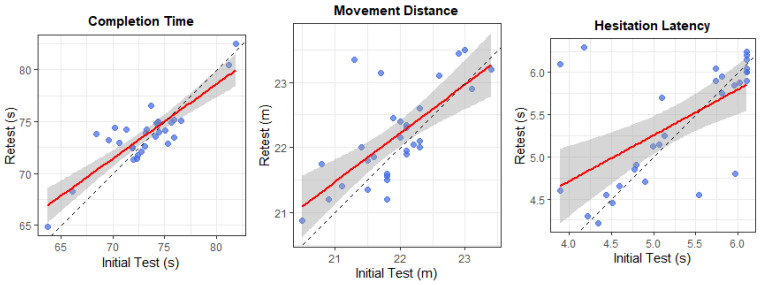
Test–retest reliability of the VRST metrics between baseline and two-week follow-up.

To assess test–retest reliability, VRST performance was reassessed after a two-week interval under the same testing conditions. ICCs showed high reliability for all VRST outcome measures except for the number of errors. Specifically, ICCs for completion time (ICC = 0.92, 95% CI: 0.86–0.96), movement distance (ICC = 0.90, 95% CI = 0.82 to 0.95), and hesitation latency (ICC = 0.91, 95% CI = 0.84 to 0.96) were all above 0.9 and statistically significant (all *p* < 0.001) (Figure 4). These values indicate excellent test–retest reliability.

## 4. Discussion

This study aimed to evaluate the validity and reliability of the VRST developed to assess inhibitory control among healthy older adults. The findings revealed that all VRST outcome measures—except for error count—were significantly correlated with the corresponding performance indicators of the traditional Stroop test. In addition, the VRST showed high test–retest reliability over a two-week interval, particularly for response time, movement distance, and hesitation latency.

The VRST was designed based on the embodied cognition framework [33], enabling the assessment of not only cognitive accuracy and response speed but also real-time physical behaviors such as hand movement and hesitation latency. In this test, participants performed a Stroop-like task in a realistic virtual context that simulated a daily activity—categorizing clothing items using a hand controller. The inclusion of kinematic data as behavioral indicators represents a shift toward more ecologically valid methods of evaluating executive function [13,34,35,36]. Unlike existing VR-Stroop paradigms that primarily translate 2D stimuli into 3D visual environments, the VRST leverages the continuous flow of information from cognition to action. Theoretically, this allows us to observe the real-time completion between target-driven and distractor-driven motor plans. Our movement-based metrics, such as distance and hesitation latency, thus offer a unique window into the temporal dynamics of inhibitory control that are often masked in discrete reaction time measures.

Previous studies have emphasized the role of physical behavior in reflecting cognitive processes [13,34,35,36]. The present results align with this body of literature, particularly in demonstrating that longer movement distances and greater hesitation latency are associated with poorer Stroop performance, suggesting that motor indicators can serve as objective proxies for cognitive inhibition. These findings support the embodied cognition perspective, which posits that cognitive processing is deeply linked to physical interactions with the environment [13,34,35,36]. This perspective is further substantiated by studies that have shown strong associations between executive function and movement kinematics during VR-based daily life tasks [13].

The positive correlation between the VRST metrics and traditional Stroop completion time is consistent with prior research demonstrating that movement efficiency is tied to better executive control [13,34,35,36]. In a related study, a virtual reality-based Stroop test called the “Stroop Room” was developed to measure participants’ physiological responses, such as heart rate and skin conductance, during the task. The findings demonstrated that the VR-based Stroop test effectively induced cognitive stress, highlighting its potential as a tool for assessing inhibitory control in immersive environments [37].

The VRST metrics provide a granular window into the temporal dynamics of inhibitory control. First, total completion time reflects the efficiency of the overall attentional control system, consistent with Sternberg’s memory scanning paradigm [27], where cognitive load directly modulates processing speed. Beyond discrete speed, the kinematic variables capture the online resolution of response completion. According to Internal Model Theory [38], the brain generates forward models to predict and execute efficient motor commands. In our task, 3D trajectory length serves as a kinematic proxy for the continuous “tug-of-war” between the goal-directed motor plan and distractor-driven interference. Even in healthy older adults, subtle inhibitory lapses in the dorsolateral prefrontal cortex (DLPFC) can manifest as increased spatial variability, as the supplementary motor area (SMA) must constantly recalibrate motor commands to correct for interference [39]. Similarly, hesitation latency is linked to proactive inhibition—the ability to withhold a response until the correct action is selected. This aligns with dual-process theory [29] and the Diffusion Decision Model [28], where the decision threshold is reached only after controlled processing overcomes the automatic response. A longer latency indicates greater neural effort within the fronto-striatal circuits to inhibit the prepotent motor plan before movement initiation. By explicitly linking these indicators to the DLPFC-SMA inhibitory circuit, the VRST demonstrates its sensitivity in detecting subtle executive fluctuations that remain latent in traditional discrete assessments.

VR further strengthens ecological validity by simulating real-world tasks and enabling standardized task delivery while capturing detailed behavioral and kinematic data that extend beyond traditional test metrics [9,13,19]. This approach is particularly valuable where subtle executive dysfunction may go undetected using conventional assessments [17,40].

On the other hand, the VRST demonstrated high test–retest reliability, suggesting that it can be used consistently over time without significant variability. This is significant given that traditional neuropsychological tests often show inconsistency due to variation in examiner delivery or participant familiarity with test materials [41]. In contrast, computerized VR assessments standardize task presentation and enable precise recording of user behavior [19]. Consequently, prior VR studies have assumed consistent measurement without formally testing for reliability [19], Nevertheless, our results provide empirical evidence for the reproducibility of VR-based cognitive assessments. One possible factor contributing to the high reliability observed in this study is the structured tutorial provided prior to testing. Ensuring that participants were comfortable with the VR interface likely minimized performance variability caused by unfamiliarity with the device. This is especially important in older adult populations, who may initially experience discomfort or hesitancy with digital tools [42]. However, it is worth noting that the error count variable demonstrated low test–retest reliability and did not show a significant correlation with traditional Stroop performance. This may be due to a limited range of error variability, possibly resulting from task simplicity or a ceiling effect commonly observed in high-functioning healthy older adults [16]. Yet, this lack of correlation does not necessarily imply a lack of sensitivity in the VRST. While traditional error counts only capture discrete failures of inhibition, VRST kinematic indicators like hesitation latency provide a more granular measure by capturing the pre-error cognitive interference and the real-time resolution of conflict during successful trials Future iterations of the VRST may benefit from incorporating adaptive difficulty levels to increase task sensitivity and better capture meaningful differences in inhibitory control.

The key strength of this study lies in its integration of embodied cognition principles into a clinically applicable assessment. By extracting movement-related behavioral data during cognitive task performance, the VRST extends beyond conventional paper-and-pencil measures. These kinematic indicators have the potential to serve as normative benchmarks for early detection of executive function decline in clinical settings. Importantly, this approach allows for the detection of subtle impairments in inhibitory control that may not yet be reflected in overt cognitive errors [43,44,45]. If applied appropriately, such VR-based methods may serve as an early warning system for executive dysfunction, allowing for timely intervention. Moreover, the VRST could be especially useful in settings where the availability of trained neuropsychological examiners is limited.

Despite its promising findings, this study has several limitations. First, this study was conducted as a preliminary investigation and therefore recruited healthy older adults with preserved cognitive function. While the findings suggest that the VRST may be informative for characterizing inhibitory control performance, this study did not directly assess clinical populations—such as individuals with mild cognitive impairment or executive dysfunction—which remains a key limitation. Accordingly, future studies should examine how VRST outcomes vary across different levels of executive functioning, including clinical samples, to clarify its broader applicability. Second, while the VR environment was designed to be intuitive, technological constraints may still limit the full realization of naturalistic affordances. From the perspective of embodied cognition, these constraints may interfere with transparency of interaction, where the technological interface should ideally become an invisible extension of the user’s body [46]. If the interaction is not fully seamless, a portion of the user’s cognitive resources may be diverted to managing the interface itself, potentially confounding the kinematic markers of inhibitory control. Although our results demonstrate that VRST kinematics still provide high validity and reliability, future refinements in hardware are necessary to minimize these external artifacts and more purely capture the fluid coupling between cognition and action. The embodied cognition approach assumes a seamless interaction between user and environment, and further refinement of VR hardware and task design is needed to enhance immersion and ecological validity. Third, this study did not account for participants’ prior experience with digital devices, which may have influenced VRST performance. Additionally, differences in controller familiarity that may vary by age were not considered and could have affected task execution. A potential sampling bias should be considered, as older adults who voluntarily participate in VR research may possess higher digital literacy or greater openness to technology than the general population. This self-selection bias could result in superior VRST performance, potentially limiting the generalizability of our findings to those with low technological familiarity. Future studies should include digital literacy as a covariate or utilize subgroup analyses to examine how prior digital experience influences the validity and reliability of VR-derived cognitive markers [42]. Future studies should control for digital literacy as a covariate or include it in subgroup analyses. Moreover, incorporating post-assessment interviews may have provided a deeper understanding of participants’ authentic reactions, attitudes toward the VRST, and critical reflections on the testing experience, which could inform further development and usability optimization. Fourth, VRST error rates were not correlated with traditional Stroop errors. This limitation is primarily attributable to a ceiling effect among cognitively healthy older adults, where restricted error variability limited statistical sensitivity. To address this, future iterations should incorporate adaptive task difficulty—such as modulating stimulus presentation speeds—to elicit a broader distribution of performance. Furthermore, we acknowledge the need to employ Signal Detection Theory (SDT) metrics (e.g., sensitivity d’ and response criterion) rather than raw error counts [47]. Integrating SDT will allow for a more nuanced distinction between genuine inhibitory deficits and individual response biases, thereby enhancing the theoretical rigor and diagnostic power of the VRST. Fifth, although both the VRST and the traditional Stroop test aim to assess inhibitory control, their differences in response modality (motor vs. verbal) and stimulus presentation (object manipulation vs. word-color naming) may limit the direct comparability of their outcomes. Future studies should consider validating the VRST against modality-matched tasks to more accurately establish concurrent validity. Finally, the cross-sectional design of this study precludes assessing causal relationships or long-term cognitive trajectories. Consequently, our findings do not establish predictive validity for the conversion from healthy aging to MCI or dementia. While the VRST’s kinematic variables demonstrate strong concurrent validity, future longitudinal research is necessary to determine their prognostic utility in predicting clinical decline and identifying individuals at risk for future neurodegeneration.

## 5. Conclusions

This study demonstrated that the VRST, developed based on the principles of embodied cognition, is a valid and reliable tool for assessing inhibitory control in cognitively healthy older adults. The VRST outcomes—including task completion time, hand movement distance, and hesitation latency—were significantly correlated with traditional Stroop test performance, providing evidence for concurrent validity. Moreover, the VRST exhibited high test–retest reliability over a two-week interval, supporting its consistency and reproducibility.

Unlike conventional paper-and-pencil tests, the VRST offers an ecologically grounded assessment by integrating real-time physical interaction into a simulated daily living task. This allows for the measurement of subtle motor and cognitive features that may not be captured through traditional assessments alone. By capturing nuanced behavioral metrics such as hesitation latency and movement efficiency, the VRST may be useful for detecting subtle variations or declines in executive functioning within healthy aging populations.

However, it is important to note that since this study exclusively recruited cognitively healthy individuals to establish a normative baseline, these findings cannot be directly generalized to clinical populations with overt cognitive impairments. Rather than serving as a standalone diagnostic or screening tool, the present findings support the VRST as a complementary assessment approach for characterizing executive function performance in healthy older adults. Future research should extend this approach to clinical populations, such as individuals with mild cognitive impairment or early-stage dementia, to validate its diagnostic sensitivity across various cognitive statuses and to establish broader clinical applicability.

## Figures and Tables

**Table 1:** Comparison of research scope and data utilization between the previous and current study.

Comparison Item	Previous Study	Current Study
Research focus	Clinical discrimination (mild cognitive impairment vs. Healthy)	Psychometric validation (Reliability & Validity)
Data subset	Combined cohort	Healthy subset only
Variables used	Same kinematic metrics	
Measurement	Single session	Repeated session
Primary analysis	ROC curves, AUC	ICC, Pearson’s correlation
Key contribution	Demonstrated utility for diagnosis	Established reliability and validity of the tool

ROC: Receiver Operating Characteristics, AUC: Area Under the Curve, ICC: Intraclass Correlation.

**Table 2:** General and clinical characteristics of participants (N = 224).

Variables	Mean (Standard Deviation)
Age (year)	71.51 (2.89)
Sex (male/female)	103/121
Education period (year)	5.94 (2.19)
Grooved pegboard test, preferred hand (s)	73.39 (14.16)
MMSE-K (point)	26.77 (1.22)

MMSE-K: Korean version of Mini-Mental State Examination.

**Table 3:** Performance on VR-based and traditional Stroop tests (N = 224).

Variables		Mean (Standard Deviation)
VRST	Completion time (s)	72.65 (3.15)
	Error count	3.32 (2.11)
	Movement distance in XYZ (cm)	22.40 (1.33)
	Hesitation latency (s)	5.33 (0.70)
Conventional Stroop test	Completion time (s)	71.67 (6.03)
	Error count	5.47 (1.58)

VRST: Virtual reality-based Stroop test.

**Table 4:** Correlation between performance of the VR-based and traditional Stroop tests (N = 224).

Variables		Conventional Stroop Test	
		Completion Time (s)	Error Count
VRST	Completion time (s)	0.821 **	0.322
	Error count	−0.188	−0.098
	Movement distance in XYZ (cm)	0.801 ***	0.298
	Hesitation latency (s)	0.784 **	0.288

** *p* < 0.01, *** *p* < 0.001, VRST: Virtual reality-based Stroop test.

## Data Availability

The raw data supporting the conclusions of this article will be made available by the authors on request.

## Abbreviations

The following abbreviations are used in this manuscript:

HMD	Head-mounted display
ICC	Intraclass correlation coefficient
MMSE-K	Korean version of the Mini-Mental State Examination
SDT	Signal detection theory
VRST	Virtual reality-based stroop test
